# Umbilical cord haematoma in a preterm newborn

**DOI:** 10.31744/einstein_journal/2022AI0008

**Published:** 2022-08-12

**Authors:** Ana Isabel Foles, Cristina Pinto Gago, Joana Soares, Marta Aguiar, Maria Contreiras Knoblich, Madalena Lopo Tuna

**Affiliations:** 1 Centro Hospitalar de Lisboa Ocidental Lisboa Portugal Centro Hospitalar de Lisboa Ocidental, Lisboa, Portugal.; 2 Centro Hospitalar de Setúbal Setúbal Portugal Centro Hospitalar de Setúbal, Setúbal, Portugal.; 3 Hospital de Cascais Dr. José de Almeida Cascais Portugal Hospital de Cascais Dr. José de Almeida, Cascais, Portugal.

Umbilical cord haematoma (UCH) is a spontaneous bleeding in the umbilical cord caused by the rupture of umbilical vessels with blood extravasation in the Warton’s jelly. This condition is a rare complication of pregnancy (1:5000-11000 deliveries). UCH may cause fetal distress and it can be associated with stillbirth (50% of cases). For these reasons, close surveillance is required when diagnosed by antenatal ultrasound.^( 1 - 3 )^

Various risk factors have been suggested such as cord anomalies, infection, coagulation disorder, post maturity, and iatrogenic-related (amniocentesis, fetal diagnostic procedures and *in utero* transfusion).^( 4 - 6 )^

**Figure 1:**
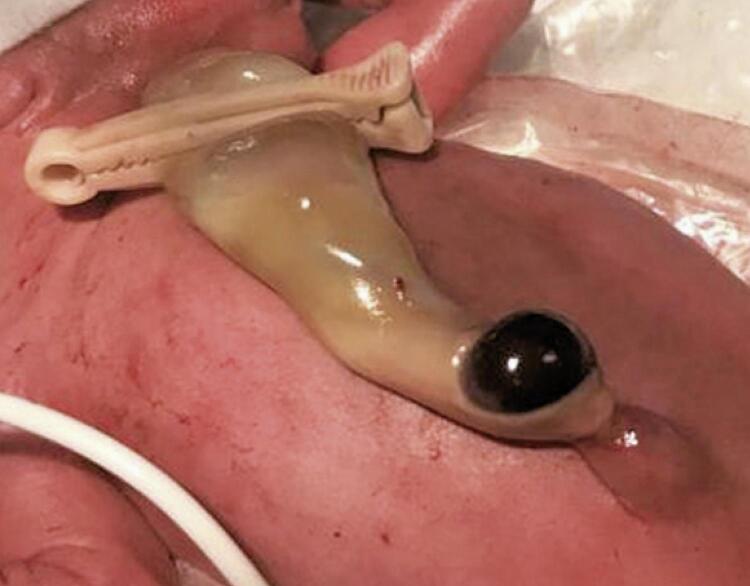
Umbilical cord haematoma right after birth

**Figure 2:**
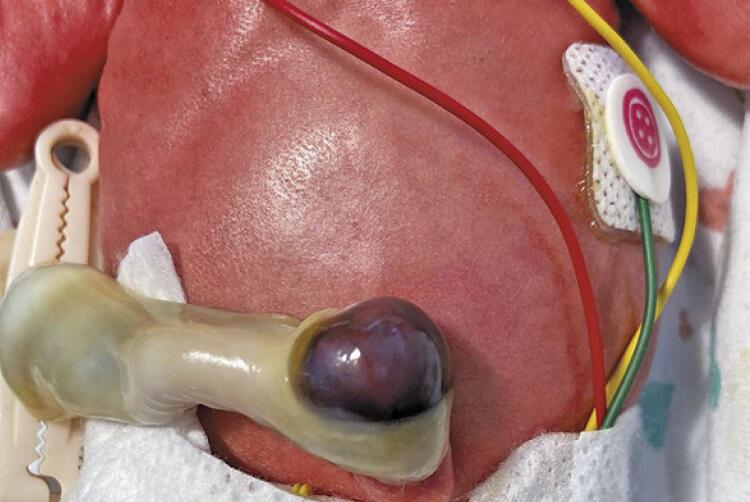
Umbilical cord haematoma on the first day of life

A 31-year-old pregnant woman was admitted at 26 weeks of gestational age with preterm premature rupture of membranes (PPROM). Pregnancy was previously uneventful, without history of trauma or *in utero* procedures. A female preterm, weighing 887g, was born at 27 weeks and 6 days, by emergent cesarian section after marginal placental abruption. At delivery a brownish-purple non-tender swelling in the umbilical cord, measuring 2x2cm, was noted ( Figure 1 and 2 ). Cord examination showed 3 vessels, without other abnormalities. Ultrasound scan excluded omphalocele and confirmed the hypothesis of UCH. Early blood tests excluded anemia, coagulation abnormalities or elevated acute inflammatory markers. Umbilical catheterization was not performed, and a peripheral vein catheterization was used instead.

**Figure 3:**
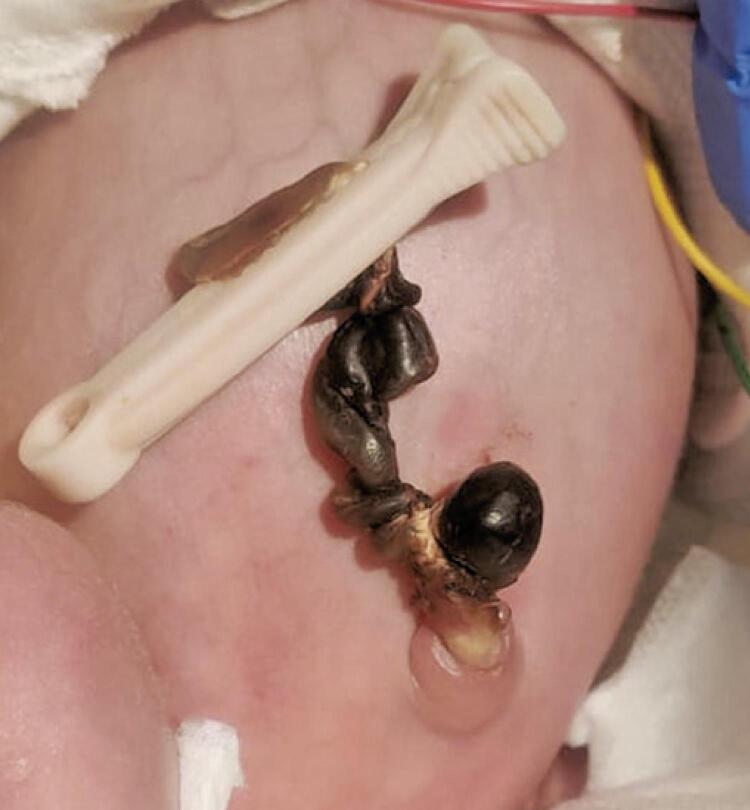
Umbilical cord haematoma on the 24th day of life

**Figure 4:**
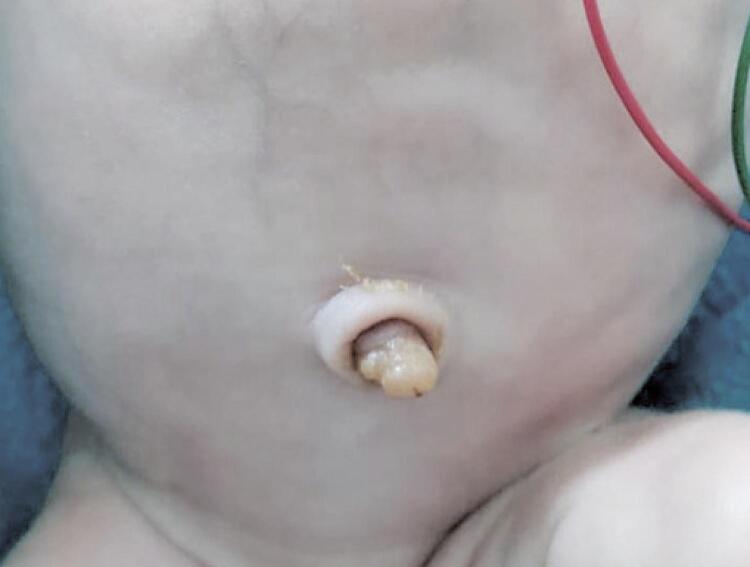
Remaining portion of the umbilical cord, after mummification and fall of the distal part of the cord (including haematoma)

The anatomopathological examination of the placenta and umbilical cord documented a well-defined saccular hematoma in the area adjacent to the cord insertion, diffuse acute chorioamnionitis, and funisitis. The newborn required systemic antibiotics for early neonatal sepsis. The umbilical cord stump evolved to mummification ( Figure 3 ), with delayed fall on 30^th^ day of life. Congenital hypothyroidism was excluded. The proximal portion of the umbilical cord remained moist ( Figure 4 ) requiring application of silver nitrate for complete regression.

Most case reports in published literature describes spontaneous UCH in full-term newborns.^( 2 , 7 - 10 )^ In our case, a spontaneous UCH was found in a preterm newborn, with history of placental abruption, PPROM and histological chorioamnionitis and funisitis. The clinical suspicion was promptly confirmed, and potential complications were excluded.

Umbilical cord haematoma is a challenging prenatal diagnosis, requiring close surveillance when diagnosed by antenatal ultrasound, as compression of umbilical vessels may cause perinatal asphyxia and stillbirth.

The umbilical cord stump delayed fall related with UCH was previously described in literature.^( 9 )^ In our case, we believe the delayed fall of the umbilical cord stump was related with the presence of the voluminous UCH associated with risk factors, such as cesarean section, prematurity, very low weight at birth, and systemic antibiotic.
